# Enhanced Accumulation of Copper and Lead in Amaranth (*Amaranthus paniculatus*), Indian Mustard (*Brassica juncea*) and Sunflower (*Helianthus annuus*)

**DOI:** 10.1371/journal.pone.0062941

**Published:** 2013-05-08

**Authors:** Motior M. Rahman, Sofian M. Azirun, Amru N. Boyce

**Affiliations:** Institute of Biological Sciences, Faculty of Science, University of Malaya, Kuala Lumpur, Malaysia; University of Nottingham, United Kingdom

## Abstract

**Background:**

Soil contamination by copper (Cu) and lead (Pb) is a widespread environmental problem. For phytoextraction to be successful and viable in environmental remediation, strategies that can improve plant uptake must be identified. In the present study we investigated the use of nitrogen (N) fertilizer as an efficient way to enhance accumulation of Cu and Pb from contaminated industrial soils into amaranth, Indian mustard and sunflower.

**Methods/Principal Findings:**

Plants were grown in a greenhouse and fertilized with N fertilizer at rates of 0, 190 and 380 mg kg^−1^ soil. Shoots, roots and total accumulation of Cu and Pb, transfer factor (TF), translocation index were assessed to evaluate the transport and translocation ability of tested plants. Addition of N fertilizer acidified the industrial soil and caused the pH to decrease to 5.5 from an initial pH of 6.9. Industrial soil amended with N fertilizer resulted in the highest accumulation of Pb and Cu (for Pb 10.1–15.5 mg kg^−1^, for Cu 11.6–16.8 mg kg^−1^) in the shoots, which was two to four folds higher relative to the concentration in roots in all the three plants used. Sunflower removed significantly higher Pb (50–54%) and Cu (34–38%) followed by amaranth and Indian mustard from industrial soils with the application of N fertilizer. The TF was <1 while the shoot and root concentration (SC/RC) ratios of Pb and Cu were between 1.3–4.3 and 1.8–3.8, respectively, regardless of plant species.

**Conclusions:**

Sunflower is the best plant species to carry out phytoextraction of Pb and Cu. In contrast, Pb and Cu removal by Indian mustard and amaranth shows great potential as quick and short duration vegetable crops. The results suggest that the application of N fertilizer in contaminated industrial soil is an effective amendment for the phytoextraction of Pb and Cu from contaminated industrial soils.

## Introduction

A major environmental concern resulting from the dispersal of industrial and urban wastes generated by human activities is the contamination of soil and its environment [1]. Soils may become contaminated by the accumulation of toxic heavy metals and metalloids through emissions from rapidly expanding industrial areas, mine tailings, disposal of high metal wastes, leaded gasoline and paints, application of fertilizers and manures, sewage sludge, pesticides, wastewater irrigation, coal combustion residues, spillage of petrochemicals, and atmospheric deposition [2], [3]. Soils are the major sink for toxic metals released into the environment by the aforementioned anthropogenic activities and unlike organic contaminants which are oxidized to carbon (IV) oxide by microbial action, most metals do not undergo microbial or chemical degradation [4], and their total concentration in soils persists for a long time after their introduction [5], although changes in their chemical forms and bioavailability are possible. The presence of toxic metals in soil can severely inhibit the biodegradation of organic contaminants by microorganisms [6]. Among various types of soil pollutants, toxic metals pollution appears to be of great concern, especially in developing countries where it has caused soil quality deterioration either by aerial deposition or waste water discharge [7].

Regardless of the origin of the metals in the soil, high levels of many metals can result in the degradation of soil fertility and poor quality of agricultural products and poses a significant threat and hazard to human, animal and ecosystem well-being [8], [9]. Metals such as Cu, Pb and zinc (Zn) are particularly important since high quantities of these metals can decrease crop production due to the risk of biomagnifications and bioaccumulation in the food chain. There is also the risk of underground and surface water contamination [10], [11]. These cannot be removed biologically and under certain circumstances can transform from one oxidation state to another which in some metals makes them less toxic [12]. Toxic metal contamination of soil may pose risks and hazards to humans and the ecosystem through direct ingestion or contact with contaminated soil, the food chain, drinking of contaminated ground water, reduction in food quality via phytotoxicity, reduction in land usability for agricultural production causing food insecurity, and land tenure problems [13], [14].

Accumulation of toxic metals in crop plants is also of great concern because it can enter the food chain [15]. As plants acquire the necessary nutrients, such as N, phosphorus (P) and potassium (K), they also take in and accumulate toxic metals such as Pb and cadmium (Cd). Ingestion of vegetables grown in soils contaminated with toxic metals has been suggested as a possible risk to human and wildlife health. These health risks will depend on the chemical composition of the contaminated soil, its physical characteristics, the type of vegetables cultivated and the consumption rate [16]. The uptake of toxic metals by plants is often influenced by plant species, growth stage, soil type, metals and environmental factors. Toxic metal concentration in the soil solution plays a critical role in controlling metal availability to plants [17]. Many findings have shown that increasing the levels of toxic metals in the soil will bring about an increased uptake by the plants. However, the availability of the toxic metal ions are influenced by various factors including soil pH, the physical and chemical properties of soil, the clay content and manganese oxide concentration [18].

In developing countries with high population density and insufficient funds available for environmental restoration, low cost and ecologically sustainable remedial options are required to restore contaminated lands so as to reduce the associated risks, make the land resource available for agricultural production, enhance food security, and scale down land tenure problems [19]. Phytoextraction was developed as a result of efforts to find a more efficient and less hazardous technique to remediate contaminated soils [20]. It involved the removal of metals by plants through uptake and accumulation into biomass [1]. Interestingly, although phytoremediation was recognized and documented by humans more than 300 years ago, its scientific study and development was not conducted until the early 1980's [21]. However, progress in making phytoextraction a practical commercial technology has been hindered by the lack of strategies to optimize plant uptake of metals [22]. Contaminated soil can be amended by conventional ways such as physical, chemical, bioremediation and phytoremediation. Unfortunately, existing remediation methods for toxic metal removal from contaminated soils are expensive and disruptive [1]. In recent years, efforts have focused on the remediation strategies that are less expensive, less destructive and more sustainable [23]–[25]. Phytoremediation, defined as the use of plants to remove pollutants from the environment, is a promising technology for the remediation of contaminated soils and perhaps for the removal of metals from contaminated soil [26]. This technology can be applied to both organic and inorganic pollutants present in soil, water and air. In this respect, plants can be compared to solar driven pumps which can extract and concentrate certain elements from the environment [27]. Phytoremediation is aesthetically pleasant, soil-microorganisms friendly, enhances diversity, and derives energy from sunlight [28]. More essentially, it is able to retain the fertility status of the soil even after the removal of toxic metals. Many plants are capable of accumulating high levels of toxic metals in the soil and are potent tools to help clean up contaminated soil [29]. The success of phytoremediation depends on the solubility of heavy metals and the ability of plant to take in and partition toxic metals to the upper plant parts [30]. Several studies have been done with chelating agents for their ability to dissolve metals and enhance the uptake of metals by plants. However these chemicals have limitations due to their negative effects on the plant and soil properties [31]–[33]. Phytoremediation has been recommended as an alternative remediation technology for polluted soil with heavy metals but is generally perceived to be too slow. The enhance accumulation of trace pollutants in harvestable plant tissues is a prerequisite for such technology to be practical. Thus, an ideal plant selection for rehabilitation of polluted soil has to be considered based on faster and higher biomass producing plants that can tolerate and accumulate heavy metals in plant parts. Relationships between toxic metals in crops and pollution of toxic metals in soils have been well documented [34]. However, current information on the phytoremediation technology using tropical species grown with nitrogen fertilizer is limited. Taking this into account a study was undertaken to investigate the impact of N fertilizer on the accumulation of Cu and Pb in industrial soils using amaranth, Indian mustard and sunflower plants.

## Materials and Methods

### Soil Management and Experimental Design

Experiments were conducted in a greenhouse at the University of Malaya, Kuala Lumpur, Malaysia from September 2010 to December 2010. Soil samples were collected from the 0–30 cm depth randomly from 10 spots within an area of recycling electronic wastes and chemicals that contained heavy metals in Senai, Johor, (1° 28′ 0″ N, 103° 45′ 0″ E) Malaysia. No specific permits were required for the described field studies and no specific permissions were required for these activities. In addition the locations were not protected and the field studies did not involve endangered or protected species. The industrial soils were sandy loam in texture and thoroughly mixed and sieved through a 2-mm mesh to obtain homogenous soil composites before they were used as the growing medium in polyethylene pots (height 18-cm × diameter 14-cm = surface area 0.095 m^2^). Each pot was filled with 2 kg of industrial soil. Amaranth, Indian mustard and sunflower were grown in industrial soil with N fertilizer at rates of 0, 190 and 380 mg kg^−1^ soil. Each plant species was tested in an individual experiment against the three levels of N fertilizer. The experiment was conducted under completely randomized design with six replications. The seeds of amaranth, Indian mustard and sunflower were sown as per treatment. Sufficient seeds were sown to ensure healthy germination. Plants were thinned after germination and five plants per pot were kept until harvesting. Prior to sowing 0.41 g and 0.82 g urea (46%N) were applied into the soils as per treatment schedule. Continuous monitoring was done to observe growth and all plants were harvested at the age of 56 d.

### Plant and Soil Analysis

Prior to planting, the initial Pb and Cu concentrations in the industrial soil were analyzed using an atomic absorption spectrometer (AAS). In industrial soil, Cu (56.2±0.09 mg kg^−1^) and Pb (40.2±0.07 mg kg^−1^) were present. The initial status of soil N in industrial soil was 26.50±0.71 mg kg^−1^. The soil pH was determined from the prepared soil suspension (1∶2.5 soil water ratios) by using a combined pH meter model 900A (Thermo Orion, Ontario, Canada) at regular 14 d interval [35]. After harvesting all plant samples were oven-dried at 72°C for 48 h. Biomass accumulation and partitioning were determined for all the plants used. Shoot and root tissues of each plant species and soils were firstly ground by using mortar and pestle prior to toxic metal analysis. For each soil and plant parts, 800 mg of the ground particle was diluted with 1 L of distilled water to produce an 800 ppm mixture. Lead and Cu concentrations in each soil and plant parts were analyzed using a PE Analyst 400 AAS.

### Calculation

The Cu and Pb transport from soil to the shoots was evaluated using the transfer factor (TF) = WC (mg kg^−1^)/TC (mg kg^−1^) where, WC = elements concentration in whole plant; TC = elements soil concentration obtained with AAS [36]. The ability for Cu and Pb translocation from roots to shoots was calculated by the translocation index (TI) % = SQ (mg per pot)/WPQ (mg per pot)×100, where, SQ = element accumulation in the shoots; WPQ = element accumulation in the whole plant (shoots+roots). Percentage removal (%) was calculated from the total concentration (TC) of elements initially present in the soil [37].

### Data Analysis

Statistical analysis was carried out by one-way ANOVA using general linear model and t-Test to evaluate significant differences between means of plant biomass, absorbed heavy metal concentrations in harvested plants at 95% level of confidence [38]. Further statistical validity of the differences among treatment means was estimated using the least significance difference (LSD) method.

## Results and Discussion

### Soil pH in Industrial Soil

Soil pH in the industrial soil was significantly affected by the application of N fertilizer regardless of plant species. From an initial pH of 6.9 the soil pH decreased significantly after the application of N fertilizer. Nitrogen fertilizer application at the rate of 380 mg kg^−1^ soil developed a more acidic soil pH during experimental period. The lowest soil pH was between 5.5–5.6, with the use of N fertilizer at rates of 190 mg kg^−1^ soil and 380 mg kg^−1^ soil at 14 d. Thereafter the soil pH increased slightly over time in both unfertilized and fertilized industrial soils regardless of plant species (Table 1). This could possibly be due to the stable saturated moisture in the soils and plants that will result in a dilution effect of the soil pH by the presence of hydroxyl ions (OH^−^) in water, particularly in the unfertilized industrial soil experiments. The change in soil pH with N fertilizer treatment probably involved more complex reactions due to the application of varying levels of N fertilizer. The initial acidic condition of fertilized industrial soil at 14 d is possibly due to the application of N fertilizer and the subsequent microbial stimulated aerobic conversion of NH_4_^+^ to NO_3_^−^ through nitrification, which will release H^+^ ions into the soil. Enhanced nitrate uptake by the plants could potentially affect soil pH as a result of OH^−^ excretion and the intake of protons (H^+^) during uptake of soil nitrate.

**Table 1:** Soil pH influenced by N fertilizer with amaranth, Indian mustard and sunflower grown in industrial soils over time.

Plant species andN fertilizer(mg kg^−1^ soil)	Days after emergence				
	0	14	28	42	56
Amaranth:					
N 0	6.9 a	6.8 a	6.8 a	6.9 a	7.0 a
N 190	6.9 a	5.6 b	6.0 b	6.2 b	6.4 b
N 380	6.9 a	5.5 b	5.8 c	6.0 c	6.2 c
Indian Mustard:					
N 0	6.9 a	6.8 a	6.9 a	7.0 a	7.0 a
N 190	6.9 a	5.6 b	6.0 b	6.3 b	6.5 b
N 380	6.9 a	5.5 b	5.8 c	5.9 c	6.2 c
Sunflower:					
N 0	6.9 a	6.8 a	6.9 a	7.0 a	7.0 a
N 190	6.9 a	5.6 b	6.0 b	6.2 b	6.4 b
N 380	6.9 a	5.5 b	5.8 c	6.0 c	6.3 c

Means followed by the same letters are not significantly different for each treatment means (*P<0.05*).

Soil pH greatly influences the availability of plant nutrients [39] and controls the solubility of heavy metals and other plant nutrients in the soil that subsequently influences the effectiveness of phytoremediation. Lead is relatively unavailable to plants when the soil pH is above 6.5 and even more so when soil phosphorus levels are high [40]. The decreased soil pH can bring about an increase in heavy metal sorption, due to the increased net negative surface charge on organic matter and soil oxides at high pH levels [41]. In the present study, adding N also acidified the industrial soil, which caused the pH decrease to 5.6 from an initial pH 6.9 within 56 d in the pot experiments. The optimal soil pH for plant growth has been documented to be around 6.4 even though; sunflower can tolerate soils ranging in from pH 5.7 to over 8.0. A pH range between 6.0 to 7.2 has been reported to be optimal for many soils [42] while optimal soil pH for amaranth and Indian mustard growth has been documented to be 6.4 [43] and 6.5 [44], respectively. Thus a balance between soil pH and heavy metal availability are required for enhance plant growth. In this study the soil pH increased from 5.8 to 6.5 after 56 d when the plants were grown with N fertilizer whilst the pH increased more than 6.5 when the plants were grown in unfertilized soil. The solubility of Cu is drastically increased at pH 5.5 [32], which are close to the ideal farmland pH of 6.0−6.5 [33]. Copper is an important essential element for plants, microorganisms, animals, and humans. The connection between soil and water contamination and metal uptake by plants is determined by many chemical and physical soil factors as well as the physiological properties of the crops.

### Dry Matter Yield

The shoot, root and total dry matter yield was affected significantly (*P*<0.001) by application of N fertilizer in all tested plant species (Table 2). The shoot, root and total dry matter yield in the plants varied depending on the plant species. All the plant species grown with N fertilizer exhibited the highest shoot, root and total dry matter yield. Sunflower produced appreciably higher shoot, root and total dry matter than amaranth and Indian mustard. The shoot dry matter was significantly higher than roots in all tested species.

**Table 2:** Dry matter yield, lead and copper accumulation in plant tissues.

Plant species and N fertilizer (mg kg^−1^ soil)	Dry matter yield (g pot^−1^)			Roots accumulate content (RQ) (mg pot^−1^)		Shoots accumulate content (SQ) (mg pot^−1^)		Whole plant accumulate content (WPQ) (mg pot^−1^)	
	Root	Shoot	Total	Pb	Cu	Pb	Cu	Pb	Cu
Amaranth:									
N 0	1.6 b	10.0 b	11.6 b	0.007 b	0.005 b	0.06 c	0.07 c	0.07 c	0.07 c
N 190	1.9 a	12.1 a	14.0 a	0.010 a	0.009 a	0.12 b	0.14 b	0.13 b	0.15 a
N 380	1.9 a	12.7 a	14.6 a	0.011 a	0.009 a	0.14 a	0.16 a	0.15 a	0.16 a
Indian Mustard:									
N 0	1.6 b	9.8 b	11.4 b	0.004 b	0.005 a	0.05 c	0.06 c	0.06 c	0.07 c
N 190	1.8 a	11.3 a	13.1 a	0.005 a	0.006 a	0.11 b	0.14 b	0.12 b	0.15 b
N 380	1.9 a	12.0 a	13.9 a	0.005 a	0.007 a	0.14 a	0.17 a	0.15 a	0.18 a
Sunflower:									
N 0	1.6 b	11.0 b	12.6 b	0.008 c	0.008 b	0.09 c	0.10 c	0.10 c	0.11 c
N 190	2.4 a	13.1 a	15.5 a	0.013 b	0.011 a	0.19 b	0.19 b	0.20 b	0.20 b
N 380	2.5 a	13.6 a	16.1 a	0.015 a	0.012 a	0.21 a	0.23 a	0.23 a	0.24 a

Means followed by the same letters are not significantly different for each treatment means (*P<0.05*).

The shoot, root and whole plant accumulate content of Pb and Cu per pot was used to estimate translocation index and all the attributes were affected significantly by N fertilizer application in all tested plant species (Table 2). All the plant species grown in industrial soils with N fertilizer at the rate of 380 mg kg^−1^ soil exhibited the highest shoot, root and whole plant accumulate content per pot. Sunflower produced significantly higher shoot, root and whole plant accumulate content than amaranth and Indian mustard. The shoots accumulate content was significantly higher than roots accumulate content in all the tested plant species.

Higher dry matter accumulation was possibly due to the greater vegetative growth resulting from higher photosynthetic activities influenced by the addition of N fertilizer. Sunflower and amaranth responded well to varying levels of N fertilizer in the present study. Indian mustard growth was slightly poorer compared to the other two species. The possible reason for this is the use of sandy loam soil in this study. Sunflower plants grown in industrial soils with N fertilizer exhibited significantly higher dry matter yield probably due to its broader leaves which would intercept more sunlight and enable higher rates of photosynthesis and as a result incorporate more carbon into the plants [45]. This was also observed in the case of Indian mustard grown in industrial soils with N fertilizer, where its broader leaves provide a greater surface area for photosynthesis compared to the same plant growing in unfertilized industrial soils. The results indicate that higher biomass accumulation is an important trait to enhance efficiency of phytoremediation. Soil amendments with the application of organic or inorganic fertilizer can promote higher biomass yield resulting in an increased heavy metal bioaccumulation capacity of the plants. Kenaf accumulated significantly higher biomass and absorbed higher amounts of Pb in fertilized soil compared to unfertilized soils [46]. Addition of fertilizers can significantly reduce the mobility of trace metals [47] but increases the amount of trace metals in plant shoots because of the higher total biomass [48]. The application of N and P enhanced the growth of shoot and root in Indian mustard and its subsequent ability to remove Cu from contaminated soil [49]. While N fertilizer combined with EDTA facilitated the phytoremediation efficiency of sunflower in Pb-polluted soil [50], high-dose of application of N fertilizer had an influence on the accumulation of Pb in edible parts of radish, carrot and potato. All these plants had higher concentrations of Pb when contaminated soils were treated with N fertilizer compared to untreated soils [51].

### Nitrogen, Lead and Copper Accumulation

The N uptake in all the three plant species studied were significantly higher (*P*<0.01) in industrial soil amended with N fertilizer compared to unfertilized industrial soils (Table 3). All the plant species recorded higher N uptake (64.6−75.8 mg g^−1^) in industrial soil with N application compared to unfertilized industrial soils (45.8−47.8 mg g^−1^). The shoot and root N were also significantly higher in industrial soil with N fertilizer. The shoot N was significantly higher than root N irrespective of plant species.

**Table 3:** Nitrogen, lead and copper concentration in plant tissues.

Plant species and N fertilizer (mg kg^−1^ soil)		N (mg g^−1^)			Pb (mg kg^−1^)			Cu (mg kg^−1^)		
		Root (RC)	Shoot (SC)	Total	Root (RC)	Shoot (SC)	Total	Root (RC)	Shoot (SC)	Total
Amaranth:										
N 0		16.8 b	29.2 b	46.0 b	4.8 b	6.2 b	11.0 b	3.1 b	6.6 b	9.7 b
N 190		21.9 a	43.3 a	65.2 a	5.5 a	10.5 a	16.0 a	4.5 a	11.6 a	16.1 a
N 380		22.6 a	44.4 a	67.0 a	5.7 a	11.2 a	16.9 a	4.7 a	12.5 a	17.2 a
Indian Mustard:										
N 0	16.5 b		29.3 b	45.8 b	2.5 b	5.5 b	8.0 b	2.9 b	6.3 b	9.4 b
N 190	20.6 a		44.0 a	64.6 a	2.6 ab	10.1 a	12.7 a	3.5 a	12.2 a	15.7 a
N 380	21.1 a		45.3 a	66.4 a	2.7 a	11.6 a	14.3 a	3.8 a	14.3 a	18.1 a
Sunflower:										
N 0	17.0 b		30.8 b	47.8 b	5.2 b	8.5 b	13.7 b	5.1 a	9.2 b	14.3 b
N 190	23.7 a		50.7 a	74.4 a	5.8 a	14.2 a	20.0 a	4.6 b	14.5 a	19.1 a
N 380	24.3 a		51.5 a	75.8 a	6.1 a	15.5 a	21.6 a	4.7 b	16.8 a	21.5 a

Means followed by the same letters are not significantly different for each treatment means (*P*<0.05).

In the present study, the total extracted Pb and Cu content varied depending on the plant species. Both Pb (20.0−21.6 mg kg^−1^) and Cu (19.1−21.5 mg kg^−1^) uptake by sunflower grown in industrial soil with N was significantly higher than in unfertilized industrial soil (Table 3). Indian mustard and amaranth also recorded significantly higher Pb and Cu uptake when grown with N fertilizer. Both Indian mustard and amaranth recorded higher Cu (15.7−18.1 mg kg^−1^ for Indian mustard; 18.7−21.4 mg kg^−1^ for amaranth) than Pb uptake (12.7−14.3 mg kg^−1^ for Indian mustard; 16.0−16.9 mg kg^−1^ for amaranth) from industrial soil with N fertilizer. Lead and Cu uptake by the different plant species studied were in the following order: sunflower>amaranth>Indian mustard for both unfertilized and N fertilized industrial soils. Lead and Cu concentration in shoots and roots of all the plant species studied were significantly higher (*P*<0.05) in industrial soil with N application than in unfertilized industrial soil (Table 3). Regardless of plant species and soils used, Pb and Cu concentration in the shoots were two to three folds higher than in the roots. All tested parameters showed identical results between N fertilizer levels.

In all the plant species, relatively more metal accumulated in the shoots than in the roots where it was observed that the shoot and root concentration ratio (SC/RC) in all the plant species studied for Pb was >1, suggesting that Pb translocation from root to shoot was remarkably higher and shoots acted as a sink for Pb (Table 4). Indian mustard recorded remarkably higher SC/RC ratio (>3) when grown with N fertilizer. The SC/RC ratio (>1) showed that the root translocation of Pb and its partitioning from root to shoot had a positive influence on the plant species.

**Table 4:** Lead and copper removal, shoot and root concentration ratio, translocation index and transfer factor.

Plant species andN fertilizer (mg kg^−1^ soil)		Shoot and root concentration(SC/RC) ratio		Translocation index (TI)		Transfer Factor (TF)		Removal (%)	
		Pb	Cu	Pb	Cu	Pb	Cu	Pb	Cu
Amaranth:									
N 0		1.3 b	2.1 b	89.0 b	93.0 b	0.3 b	0.2 b	27.4 b	17.2 b
N 190		1.9 a	2.6 a	92.0 a	94.3 a	0.4 a	0.3 a	39.8 a	28.6 a
N 380		2.0 a	2.7 a	92.9 a	94.7 a	0.4 a	0.4 a	42.0 a	30.6 a
Indian Mustard:									
N 0	2.2 b		2.2 b	93.1 b	93.0 b	0.2 b	0.2 b	19.9 c	16.4 c
N 190	3.9 a		3.5 a	96.1 a	95.6 a	0.3 ab	0.3 a	31.6 b	27.9 b
N 380	4.3 a		3.8 a	96.4 a	96.0 a	0.4 a	0.3 a	35.6 a	32.2 a
Sunflower:									
N 0	1.6 b		1.8 b	91.8 b	92.5 b	0.3 b	0.3 b	34.1 c	25.4 c
N 190	2.4 a		3.2 a	93.0 a	94.5 a	0.5 a	0.3 b	49.8 b	34.0 b
N 380	2.5 a		3.6 a	93.3 a	95.1 a	0.5 a	0.4 a	53.7 a	38.3 a

Means followed by the same letters are not significantly different for each treatment means (*P*<0.05).

Copper uptake in all the plant species resulted in a relatively higher concentration of Cu in the shoots compared to the roots (Table 3). Indian mustard exhibited appreciably higher SC/RC ratio (>3) followed by sunflower when industrial soil was fertilized with N. The SC/RC ratio of all plants species was >1, suggesting that Cu translocation from root to shoot was appreciable. Considering both Pb and Cu, the higher translocation index (TI) and transfer factor (TF) were obtained, in increasing order, in amaranth, mustard and sunflower with N amended soil (Table 4). For both Pb and Cu, the highest TI was obtained in Indian mustard and sunflower. Despite the high TI observed for Pb and Cu in all the species tested, the TF, which reflects the plant’s capacity to transport metals from soil to shoots, was much higher for Pb than for Cu. Such behavior can probably be attributed to high Pb mobility in the soil due to the decreasing soil pH.

The results obtained showed that sunflower removed 49.8−53.7% of Pb 34−38.3% of Cu from industrial soil with N fertilizer compared to 34.1% of Pb and 25.4% of Cu from unfertilized industrial soil (Table 4). Amaranth removed 39.8−42% of Pb and 28.6−30.6% of Cu while Indian mustard removed 31.6−35.6% of Pb and 27.9−32.2% of Cu from industrial soil with N fertilizer. The toxic metal removal efficiency by sunflower was 16−20% higher for Pb and 9−13% higher for Cu with the application of N fertilizer in industrial soils compared to unfertilized industrial soil. Similarly Pb removal efficiency by Indian mustard and amaranth was 12−16% and 13−15% higher, respectively. Copper removal efficiency by Indian mustard and amaranth was 11−15% and 11−14% higher in industrial soil with N fertilizer compared to unfertilized industrial soil.

A comparison of the metal content of the soil used in this study with others reported in the literature confirmed that it should be considered as a contaminated soil. The amounts of Cu (56.2±0.09 mg kg^−1^) and Pb (40.2±0.07 mg kg^−1^) recorded were above the maximum content commonly found in soils of industrial areas in Johor, Malaysia. Background concentrations of Pb that occur naturally in surface agricultural soils in the United States average 10 parts per million (ppm) with a range of 7 to 20 ppm [52]. Soils with Pb levels above this range are primarily the result of Pb contamination. The Canadian Council of Ministers of the Environment interim soil assessment criterion for Cu was set at 30 mg kg^−1^ [53]. However there is no reference value is available for Cu or Pb from the local environmental agency in Malaysia. The shoot and root ratios were calculated in order to evaluate the translocation of the elements inside the plant, from the roots to the shoots, and its potential accumulation in the biomass (Table 4). The SC/RC ratio values are important to estimate the potential of a plant for phytoremediation purposes. One of the selection criteria for hyperaccumulator’s identification is the leaf’s metal concentration. Accordingly a hyperaccumulator should concentrate more than 1,000 mg kg^−1^ of Ni, Pb or Cu and more than 100 mg kg^−1^ of Cd [54]. Sunflower exhibited the highest concentration of Pb in shoots followed by amaranth, while Indian mustard was the lowest for both the metals considered (Table 3). However, according to the criteria already presented, none of the plants species tested would be classified as hyperaccumulator for Cu or Pb. This contradicts with the hyperaccumulator characteristic of sunflower where the SC/RC ratio should be >1. In this study all the tested plant species achieved this target although they could not achieve a leaf metal’s concentration of 1,000 mg kg^−1^ for Pb or Cu. A possible reason for this is the short experimental period (56 d) where sunflower did not reach maturity. As a result, the leaf metal’s concentration in sunflower was <1,000 mg kg^−1^ compared to that reported in other studies. Another possible explanation might be the use of pot experiments, which could have limited plant development due to the small volume of soil used. Furthermore the immobilization of elements by root adsorption may have had some influence. This mechanism prevents element translocation from roots to shoots in some species working as a defense barrier, decreasing the phytoextraction potential of such species since only a small portion of ions associated with the roots are effectively absorbed [21].

Sunflower is able to secrete organic acids which acidify the rhizosphere and increases the solubility of toxic metals like Pb and Cu [55]. The N fertilized plants showed higher biomass and potential to facilitate phytoextraction process. Sunflower absorbed appreciably higher amounts of Pb than Indian mustard and amaranth in industrial soil with N fertilizer due to its hyperaccumulator characteristic [48]. In this study all the plant species contained relatively higher concentration of Pb in the shoots compared to roots as high metal concentrations are in available form in acidic soil due to the decreased soil pH. It is well known that Pb is an immobile metal in soil, since it readily forms a precipitate because of its low aqueous solubility within the soil matrix, and in many cases it is not readily bioavailable. In addition, many plants retain Pb in their roots via sorption and precipitation with only minimal transport to the above ground harvestable plant portions [56], [57]. The leafy vegetables like Indian mustard and amaranth are expected to absorb more Cu compared to Pb due to the physiological role of Cu in plants [17]. The SC/RC ratio for Cu uptake were >1 for all the plants which proved that Cu partitioning was performed from roots to shoots. The high values of SC/RC ratio (>1) implies that plant shoots acts as sink for the absorption of Cu. The result of this study suggests that application of inorganic N fertilizer could boost the biomass accumulation of plants and subsequently phytoremediation efficiency. However, careful measures have to be taken during fertilizer application to avoid anthropogenic source of heavy metal entering the soil and the human food chain. In the literature toxicity in plants is reported in literature to occur when Cu is found in the range of 20−100 mg kg^−1^, and Pb in the range of 30−300 mg kg^−1^ [58], [59]. In this study the accumulations of Pb and Cu in all the tested plant species were below the toxic levels (Table 2).

The most serious source of exposure to soil Pb is through direct ingestion of contaminated soil or dust. In general, plants do not absorb or accumulate Pb. However, in soils with a high Pb content, it is possible for some Pb to be taken up. Studies have shown that Pb does not readily accumulate in the fruiting parts of vegetable and fruit crops (e.g., corn, beans, squash, tomatoes, strawberries, and apples). Higher concentrations are more likely to be found in leafy vegetables (e.g., lettuce) and on the surface of root crops (e.g., carrots). Generally, it has been considered safe to use garden produce grown in soils with total Pb levels less than 300 ppm. The risk of Pb poisoning through the food chain increases as the soil Pb level rises above this concentration. Even at soil levels above 300 ppm, most of the risk is from Pb contaminated soil or dust deposits on the plants rather than from uptake of Pb by the plant [40].

### Correlation Analysis

There were significantly positive relationships between the accumulation of Pb in the three plant species grown in the contaminated industrial soil and the dosage of the N fertilizer applied to the industrial soil (Table 5). The slopes indicated that for each 1.0 mg N application in industrial soil, 0.017 to 0.022 mg of Pb was taken up into the plant tissues (Table 5). There was also a positive correlation between N fertilizer rate and Cu uptake in the three plant species. The slopes indicated that for each 1.0 mg N application in industrial soil, 0.020 to 0.025 mg of Cu was taken up into the plant tissues (Table 5). The regression equation showed that Pb uptake was higher in sunflower while Cu uptake was higher in Indian mustard among three plant species. A strong positive relationship was observed between the accumulation of Pb and biomass (r = 0.99), Cu and plant biomass accumulation (r = 0.99) in the three plant species growing in contaminated industrial soils (Table 5).

**Table 5:** Regression equation, correlation coefficient (r) and coefficients of determination (R^2^) of different parameters.

Regression equation	Correlation coefficient (r)	Coefficients of determination (R^2^)
Relationship between N fertilizer and biomass (B):		
Y_B_ (Amaranth) = 0.011X_1_+15.78	0.97**	R^2^ = 0.939
Y_B_ (Indian mustard) = 0.013X_1_+14.857	0.95**	R^2^ = 0.909
Y_B_ (Sunflower) = 0.011X_1_+17.044	0.95**	R^2^ = 0.903
Relationship between biomass and Pb uptake:		
Y_Pb_ (Amaranth) = 1.519X_2_−12.46	0.99**	R^2^ = 0.9899
Y_Pb_ (Indian mustard) = 1.504X_2_−13.849	0.99**	R^2^ = 0.9962
Y_Pb_ (Sunflower) = 2.065X_2_−20.798	0.99**	R^2^ = 0.9996
Relationship between biomass and Cu uptake:		
Y_Cu_ (Amaranth) Cu = 1.935X_2_−20.167	0.99**	R^2^ = 0.9887
Y_Cu_ (Indian mustard) = 2.112X_2_−21.504	0.99**	R^2^ = 0.9938
Y_Cu_ (Sunflower) = 1.790X_2_−15.705	0.99**	R^2^ = 0.9748
Relationship between N fertilizer and Pb uptake:		
Y_Pb_ (Amaranth) = 0.017X_1_+11.593	0.94**	R^2^ = 0.883
Y_Pb_ (Indian mustard) = 0.018X_1_+8.4324	0.97**	R^2^ = 0.941
Y_Pb_ (Sunflower) = 0.022X_1_+14.37	0.96**	R^2^ = 0.913
Relationship between N fertilizer and Cu uptake:		
Y_Cu_ (Amaranth) = 0.021X_1_+10.468	0.94**	R^2^ = 0.879
Y_Cu_ (Indian mustard) = 0.025X_1_+9.767	0.97**	R^2^ = 0.949
Y_Cu_ (Sunflower) = 0.020X_1_+14.614	0.99**	R^2^ = 0.975

X_1_ = N fertilizer rate; X_2_ = biomass;

** significant at 0.01 level of probability.

Lead and Cu concentration were positively correlated to plant biomass due to the increased plant tissue to accumulate more heavy metals. Furthermore the higher biomass indicates that plants were bigger and probably possessed higher absorption rate so that more heavy metal uptake took place [46]. The strong positive correlation between plant biomass and plant N content was due to the positive effect of N fertilizer application. As a result, the higher biomass of plants enhanced a greater absorption of more Pb and Cu and countered the effects of heavy metal immobilization [50]. The plant N content directly or indirectly affected the heavy metal concentrations in the plants, by boosting the biomass of the plants and subsequently their ability to accumulate more heavy metals. This positive correlation suggests that inorganic or other types of fertilizers, such as organic fertilizers, can be used to increase the biomass of plants and their capacity to accumulate heavy metals from the soil.

### Conclusions

The Pb and Cu uptake ability of sunflower was appreciably greater than Indian mustard and amaranth. There was a positive relationship between N fertilizer application on plant growth and their ability to absorb Pb and Cu. Nitrogen fertilizer boosted growth parameters increasing root and shoot dry matter significantly, allowing a higher uptake of toxic metals by plants grown in fertilized industrial soil. Nevertheless, careful measures must be put in place to ensure that excessive N fertilizers are not applied to cause uncontrolled rapid above ground growth resulting in weaker plants. It is suggested that phosphorus be added to boost root growth to balance the high shoot growth in the presence of N fertilizer. In this study, sunflower was the best plant species to carry out phytoextraction of Pb and Cu due to its hyperaccumulator characteristic. Both Indian mustard and amaranth also showed Pb and Cu removal potential, as quick and short duration vegetable crops. This provides the opportunity to grow Indian mustard and amaranth several times on a piece of land to remove both Pb and Cu from contaminated soil. However, the results of the present study showed that N fertilizer application in industrial soil can lead to negative consequences on indigenous soil biota in acidic soils due to its impact on soil pH. It clearly shows the need to consider the soil characteristics of polluted soil before its remediation.
